# Hops (*Humulus lupulus*) Extract Enhances Redox Resilience and Attenuates Quinolinic Acid-Induced Excitotoxic Damage in the Brain

**DOI:** 10.3390/nu18010152

**Published:** 2026-01-02

**Authors:** Daniela Ramírez Ortega, Erick R. Hernández Pérez, Montserrat Gutiérrez Magdaleno, Karla F. Meza-Sosa, Lucia Pineda Calderas, María José Álvarez Silva, Gustavo I. Vázquez Cervantes, Dinora F. González Esquivel, Wendy Leslie González Alfonso, Javier Angel Navarro Cossio, Paulina Ovalle Rodríguez, Itamar Flores, Alelí Salazar, Saul Gómez-Manzo, Benjamín Pineda, Verónica Pérez de la Cruz

**Affiliations:** 1 Neurobiochemistry and Behavior Laboratory, National Institute of Neurology and Neurosurgery “Manuel Velasco Suárez”, Mexico City 14269, Mexico; danielaramirez@innn.edu.mx (D.R.O.); mc20hepe4999@facmed.unam.mx (E.R.H.P.); goshitomgm@ciencias.unam.mx (M.G.M.); karla.meza@innn.edu.mx (K.F.M.-S.); luciapineda@exalumno.unam.mx (L.P.C.); mj.alvarez@lasallistas.org.mx (M.J.Á.S.); dinora.gonzalez@innn.edu.mx (D.F.G.E.); paulina.ovalle@innn.edu.mx (P.O.R.); 2 Neuroimmunology Laboratory, National Institute of Neurology and Neurosurgery “Manuel Velasco Suárez”, Mexico City 14269, Mexico; gustavo.vazquez@innn.edu.mx (G.I.V.C.); wendy.gonzalez@innn.edu.mx (W.L.G.A.); javier_15@ciencias.unam.mx (J.A.N.C.); itamar.flores@innn.edu.mx (I.F.); aleli.salazar@innn.edu.mx (A.S.); 3 Laboratorio de Bioquímica Genética, Instituto Nacional de Pediatría, Secretaría de Salud, Mexico City 04530, Mexico; saulmanzo@ciencias.unam.mx

**Keywords:** redox resilience, hops, neurotoxicity, neuroprotection, excitotoxicity

## Abstract

Background: *Humulus lupulus* (Hops) possesses a diverse array of bioactive compounds with reported antioxidant, anti-inflammatory, antibacterial, and neuroprotective properties. However, most studies have focused on isolated components, whose purification is costly and yields limited quantities. Objective: In this study, we aimed to evaluate whether a complete Hops extract could exert antioxidant and neuroprotective effects. Methods: First, the ability of Hops extract’s free radical scavenging capacity against superoxide, hydroxyl radical, and peroxynitrite was discovered using combinatorial chemical assays. Moreover, the used Hops extract prevented both DNA and protein degradation induced by hydroxyl radicals. Next, rats were orally administered with three different doses of Hops extract (10, 15, and 20 mg/kg/day) for 7 consecutive days. Results: Ex vivo analyses of brain tissues revealed that Hops pre-treatment attenuated FeSO_4_-induced lipid peroxidation, increased the GSH/GSSG ratio and downregulated both glutathione peroxidase and reductase activities. Additionally, the expression of the nuclear factor erythroid 2-related factor (*Nrf2*) gene was significantly elevated in the striatum of Hops-treated animals. To further explore neuroprotection, we evaluated the effect of Hops (15 mg/kg/day) in an in vivo model of excitotoxicity induced by quinolinic acid (QUIN). Pre-treatment with the Hops extract reduced QUIN-induced circling behavior, increased the translocation of NRF2 to the nucleus and decreased apoptosis in the striatum. Conclusion: These findings suggest that the whole Hops extract enhances redox resilience in the brain and confers protection against oxidative and excitotoxic insults.

## 1. Introduction

*Humulus lupulus* L., commonly known as Hops, is a perennial climbing plant that belongs to the *Cannabaceae* family, and it is primarily cultivated because of its female inflorescences (cones), which are widely used in the brewing industry due to their characteristic bitterness and scent. Beyond their industrial application, Hops have been widely used in traditional medicine, including their use in Traditional Chinese Medicine for the treatment of various diseases, while in Ayurvedic and European phytotherapy, they are used to manage menopausal symptoms, insomnia, gastrointestinal disturbances, and nervous tension [1,2,3].

In recent years, scientific interest has grown in the pharmacological properties of Hops, largely attributed to specific types of bioactive compounds such as prenylflavonoids (e.g., xanthohumol, isoxanthohumol, and 8-prenylnaringenin) and bitter acids (α- and β-acids). In this sense, 2–20% of Hop’s dry weight accounts for bitter acids, while around 2–6% for polyphenols, depending on the plant variety and the extraction methods used [4]. Numerous studies have reported that these compounds possess antioxidant, anti-inflammatory, neuroprotective, estrogenic, antimicrobial, and even antitumoral properties [2,5,6,7,8,9,10,11,12,13,14].

Among them, xanthohumol is the most studied prenylflavonoid, which exerts potent antioxidant activity through both direct free radical scavenging and indirect activation of the nuclear factor erythroid 2-related factor (NRF2) signaling pathway, which leads to the transcription of antioxidant defense genes including the heme oxygenase 1 (*Hmox1*), NAD(P)H quinone dehydrogenase 1 (*Nqo1*), gamma-glutamyl cysteine ligase (*Gclc*), thioredoxin (*Txn*) and thioredoxin reductase (*Trxr1*) [15,16]. In consequence, this activation enhances the intracellular antioxidant capacity, which results in increased levels of reduced glutathione (GSH), a well-known antioxidant. Moreover, xanthohumol exhibits anti-inflammatory effects in immune cells by reducing TLR4 levels, inhibiting NF-κB signaling, and downregulating the level of pro-inflammatory cytokines such as IL-6 and TNF-α [16,17]. In the context of neurodegeneration, these properties translate into neuroprotective effects in multiple in vivo models. For instance, xanthohumol has been shown not only to reduce amyloid plaque aggregation, but also to improve cognitive function in transgenic murine models of Alzheimer’s disease [18,19,20]. In addition, while xanthohumol reduces reactive oxygen species (ROS), it also improves motor deficits in 6-hydroxydopamine-induced Parkinsonian rodents [21,22], it decreases both the degeneration of spinal motoneurons and the progression of amyotrophic lateral sclerosis in superoxide dismutase 1 (*Sod1*) mutant animals [23].

Alongside prenylflavonoids, particularly α-acids, they also contribute to the bioactivity of Hops. In this sense, they exhibit anti-inflammatory and antioxidant properties, including the capacity to shift microglial activation from a pro-inflammatory to an anti-inflammatory phenotype, thereby reducing cytokine production and promoting neuronal survival in neurodegenerative conditions [24,25].

Despite this body of evidence, it is relevant to note that most of these studies have been based on isolated compounds, obtained via expensive and technically demanding purification techniques such as pressurized liquid extraction or chromatography [26,27,28]. These constraints limit the feasibility of translating preclinical findings into cost-effective and scalable clinical strategies. Considering this, we hypothesized that the use of complete Hops extract could offer a practical alternative while still retaining key biological activities and the synergy among various bioactive components that enhance its therapeutic efficacy. Therefore, the present study aimed to evaluate the antioxidant and neuroprotective potential of a whole Hops extract. First, the Hops extract was assessed for its free radical scavenging capacity against superoxide, hydroxyl, and peroxynitrite, using combinatorial chemistry assays. To extend these findings in an in vivo model, three subchronic oral doses of the Hops extract used (10, 15, and 20 mg/kg/day) were administered to rats, and their impact on both the brain redox state, particularly the GSH/GSSG ratio, and the expression of the *Nrf2* gene in different brain regions was evaluated. Finally, whether the pre-treatment with Hops could enhance resistance to ex vivo oxidative stress by providing in vivo protection against quinolinic acid (QUIN)-induced excitotoxicity was tested.

## 2. Materials and Methods

### 2.1. Reagents

Nicotinamide adenine dinucleotide reduced (NADH), potassium nitrite (KNO_2_), hydrogen peroxide (H_2_O_2_), deoxyribose, diethylene triamine pentaacetic acid (DTPA), ferric chloride (FeCl_3_), thiobarbituric acid (TBA), dichlorofluorescein diacetate (DCFH-DA), ethylenediaminetetraacetic acid (EDTA), apomorphine, ascorbic acid, bovine serum albumin (BSA), trichloroacetic acid (TCA), hydrochloride acid (HCl), O-pthaldehyde (OPA) were all obtained from Sigma Chemical Company (St. Louis, MO, USA). Hops extract was obtained from HopHaze^®^ (BarthHass) (Nuremberg, Germany). All other reagents were of reactive grade and were obtained from known commercial suppliers.

### 2.2. Chemical Combinatory Assays

#### 2.2.1. Superoxide Anion (O_2_•^−^) Scavenging Assay

The ability of the Hops extracts to scavenge superoxide (O_2_•^−^) was evaluated by measuring the reduction of nitroblue tetrazolium (NBT) to a purple-colored formazan, a reaction triggered by O_2_•^−^. Superoxide generation was induced via a non-enzymatic system composed of phenazine methosulfate (PMS) and nicotinamide adenine dinucleotide (NADH). The scavenging assay involved mixing 20 mM HEPES buffer (pH 7.4), 196 mM nicotinamide adenine dinucleotide (NADH), 39.2 mM NBT, 3.92 mM PMS, and different concentrations of Hops (0–1 mg/mL) in a microplate. Optical density was measured at a wavelength of 560 nm using a Sinergy H1 microplate reader (Agilent, Santa Clara, CA, USA) for 3 min with readings taken every minute. Results were presented as a percentage of total scavenging capacity.

#### 2.2.2. Hydroxyl Radical (•OH) Scavenging Assay

The •OH scavenging activity of the Hops extract was evaluated using the deoxyribose degradation method, in which •OH radicals are generated via a Fenton-like reaction catalyzed by Fe^3+^-EDTA in the presence of hydrogen peroxide (H_2_O_2_). This method has been widely used to assess scavenging capacity [29,30]. The reaction mixture contained ascorbic acid (0.2 mM), FeCl_3_ (0.2 mM), EDTA (0.208 mM), H_2_O_2_ (1 mM), deoxyribose (0.56 mM) in a phosphate buffer (20 mM, pH 7.4) and different concentrations of Hops (0–1 mg/mL). Samples were incubated at 37 °C for 1 h, followed by the addition of 1 mL of thiobarbituric acid (TBA, 0.365%, trichloroacetic acid (TCA, 15%), hydrochloric acid (HCl, 2%), and deferoxamine (DFO, 0.066%). Reaction mixtures were then boiled for 10 min, immediately cooled on ice, and then, absorbance was measured at 532 nm wavelength using a Sinergy H1 microplate reader (Agilent, Santa Clara, CA, USA). Results were expressed as a percentage of total scavenging capacity.

#### 2.2.3. Peroxynitrite (ONOO^−^) Scavenging Assay

The ONOO^−^ scavenging activity of the Hops extract was assessed by monitoring the oxidation of 2′7′-dichlorodihydrofluorescein diacetate (DCFH-DA) to its fluorescent product, dichlorofluorescein (DCF). This method provides an indirect but sensitive approach to quantify ONOO^−^-scavenging capacity. ONOO^−^ was synthesized as previously described by Beckman et al. [31], and its concentration was spectrophotometrically determined using a molar extinction coefficient of 1670 M^−1^cm^−1^ at a wavelength of 302 nm. The assay mixture included 1 mM diethylenetriaminepentaacetic acid (DTPA), 1.75 mM DCFH-DA, 35 µM ONOO^−^, and different concentrations of Hops extract (0–1 mg/mL). The mixture was prepared in black 96-well plates, and optical density was determined at 500 nm wavelength every minute for 3 min using a Sinergy H1 microplate reader (Agilent, Santa Clara, CA, USA). The 100% DCFH-DA oxidation by ONOO^−^ or 0% scavenging capacity was used to determine the percentage of ONOO^−^ scavenging ability; the DCFH-DA oxidation percentages were converted to Hops removal capacity percentages using a tube with 100% DCFH-DA oxidation as a reference [29]. ONOO^−^ concentrations were determined before each experiment. Results were expressed as a percentage of total scavenging capacity.

#### 2.2.4. Oxidative Protein Degradation Assay

The protective effect of Hops extract against hydroxyl radical-induced protein degradation was evaluated using bovine serum albumin (BSA) as a model substrate, based on a previously reported method [32]. The ●OH-generating system included: ascorbic acid (1.6 mM), EDTA (0.8 mM), and ferrous ammonium sulfate (NH_4_)_2_Fe(SO_4_)_2_. BSA (2mg/mL) was mixed with the reaction mixture, different concentrations of Hops (0–1000 µM), and H_2_O_2_ were added to initiate OH• production, and the mixture was incubated at room temperature for 1 h. Then, 50 μL of TCA (30%) was added to stop the reaction, followed by centrifugation at 15,000 rpm for 30 min. The supernatant was discarded, and the pellet was resuspended in 500 µL of NaOH (0.1 M). To observe oxidative damage in BSA, a polyacrylamide (30%) gel was used. 50 μg of protein were mixed with Laemmli loading buffer 5× (10% glycerol, 2% SDS, 25 mM Tris–HCl (pH 6.8), 5% 2-mercaptoethanol, 0.1% bromophenol blue). The gel was run at 100 V for 1 h, and then, it was dyed using Coomassie blue dye (2%) overnight. Then, the gel was washed with color remover solution (methanol (40%) and acetic acid (10%) in deionized water) for 1 h. Protein band integrity was visualized under white light and quantified using the Image J software 1.54 g version (U.S. National Institutes of Health, Bethesda, MD, USA).

#### 2.2.5. Oxidative DNA Degradation Assay

To assess the protective effect of Hops extract on DNA integrity under ●OH-generating system, the same Fenton-like reaction components as the protein assays were used. The reaction mixture consisted of 50 mM phosphate buffer (pH 7.4), 120 µg of DNA, the oxidation reagents, and various concentrations of Hops extract (0–1 mg/mL). Hydroxyl radicals generated by the addition of H_2_O_2_ were incubated at room temperature for 10 min. The reaction was stopped by mixing 6x DNA loading buffer. DNA samples were loaded onto a 2% agarose gel prepared with 1× TAE buffer and 0.5 µg/mL ethidium bromide. Electrophoresis was performed at 60 V for 20 min using the blueGel^TM^ system (miniPCR bio, Cambridge, MA, USA), DNA bands were visualized using a UVP ChemStudio imaging system AnalytikJena, Upland, CA, USA). Band intensity and degradation were analyzed using ImageJ software 1.54 g version (U.S. National Institutes of Health, Bethesda, MD, USA).

### 2.3. Animals

A total of forty male Wistar rats (240–280 g) were used in this study. Animals were housed in acrylic cages (five rats per cage) under controlled environmental conditions: 50 ± 10% relative humidity, 25 ± 3 °C temperature, and a 12:12 light/dark cycle. Standard commercial chow diet and water were available ad libitum throughout the study. All procedures were conducted in strict accordance with the Guide for the Care and Use of Laboratory Animals (National Research Council, 2011), and the experimental protocol was approved by the Institutional Animal Care and Use Committee of the National Institute of Neurology and Neurosurgery (Protocol N. 120-23). All efforts were made to minimize animal suffering.

### 2.4. Hops Extract Administration

Animals were randomly assigned to four experimental groups (n = 5 per group). Control group: vehicle-treated control (received distilled water, p.o.); Hops 10: Hops extract at 10 mg/kg/day, orally; Hops 15: Hops extract at 15 mg/kg/day, orally, and Hops 20: Hops extract at 20 mg/kg/day, orally. Treatments were administered once daily via oral gavage for 7 consecutive days. Twenty-four hours after the final administration (on day 8), rats were euthanized by decapitation under appropriate anesthesia. Brains were rapidly dissected and immediately frozen in liquid nitrogen and stored at −80 °C until further analysis.

### 2.5. Lipid Peroxidation

Lipid peroxidation was quantified by measuring thiobarbituric reactive substances (TBARS), expressed as malondialdehyde (MDA) equivalents. Approximately 30 mg of brain tissue was homogenized (1:10 *w/v*) in Krebs buffer (pH 7.4) containing: 119 mM NaCl, 4.7mM KCl, 1.2 mM MgSO_4_, 2.5 mM CaCl_2_, 5mM glucose, 1.3 mM NaH_2_PO_4_, 3mM Na_2_HPO_4_. A total of 125 µL of brain homogenate was used per condition. For basal lipid peroxidation, samples were incubated in Krebs buffer alone. For the oxidative challenge, samples were incubated in the presence of 10 µM FeSO_4_ at 37 °C in a water bath for 1 h. After incubation, 200 µL of TBA reagent (0.375 g thiobarbituric acid, 15 g trichloroacetic acid, and 2.54 mL HCl in 100 mL of distilled water) was added. Samples were boiled for 15 min, cooled on ice for 5 min, and centrifuged at 9800× *g* for 5 min. The absorbance of supernatants was expressed as µmol of MDA per mg of protein.

### 2.6. GSH/GSSG Ratio

To assess the redox status, levels of reduced (GSH) and oxidized glutathione (GSSG) were measured in brain homogenates [33]. For this, 20 mg of brain tissue were homogenized in buffer A (pH 6.8), consisting of 0.1 M potassium phosphate buffer, 5 mM DTPA, 154 mM, 154 mM KCl. Homogenates were then mixed 1:1 with buffer B (40 mM HCl, 10 mM DTPA, 20 mM ascorbic acid, and 10% TCA). Samples were spun at 14,000× *g* for 20 min, and supernatants were filtered through 0.45 µm syringe filters. For GSH detection, o-phtaldehyde (OPA) was added to react with it, forming a fluorescent isoindole. For GSSG measurement, free GSH was first masked with 7.5 mM *N*-ethylmaleimide (NEM). GSSG was then reduced with 100 mM dithiothreitol (DTT) and reacted with OPA. Fluorescence was recorded using a Sinergy H1 microplate reader (Agilent, Santa Clara, CA, USA) at wavelengths of 365 nm excitation and 430 nm emission. Results were expressed as nmoles per g of tissue.

### 2.7. Glutathione Reductase (GR) and Glutathione Peroxidase (GPx) Activity

GR and GPx enzymatic activities were evaluated in brain tissue. Thirty milligrams of brain tissue were homogenized in 200 µL of phosphate buffer (50 mM, pH 7) containing 0.05% Triton X-100. Homogenates were centrifuged at 10,000× *g* at 4 °C for 30 min to obtain the supernatants. For GR activity, 16.5 µL of supernatant was incubated with a reaction cocktail containing 0.5 mM EDTA, 1 mM NADPH, and 2.5 mM GSSG, and absorbance was measured at 340 nm every minute for 3 min using a Synergy H1 microplate reader (Agilent, Santa Clara, CA, USA). For GPx activity, 25 µL of the same supernatant were incubated with a reaction cocktail containing 1 mM GSH, 0.2 mM NADPH, and 1 UmL/mL glutathione reductase, followed by the addition of 100 µL of 2.5 mM H_2_O_2_. Absorbance was recorded at 340 nm wavelength every 30 s for 5 min. Enzyme activities were expressed as units per milligram of protein per minute.

### 2.8. Catalase Activity

Catalase activity was evaluated in brain homogenates prepared at a 1:10 (*w/v*) in phosphate buffer (10 mM KH_2_PO_4_ and 10 mM K_2_HPO_4_, pH 7.0). Samples were centrifuged at 10,000× *g* at 4 °C for 15 min, and the resulting supernatants were used for enzymatic analysis. The assay was conducted at 28 °C in 96-well plates. The reaction mixture consisted of 20 µL of the supernatant, 180 µL of phosphate buffer, and 10 µL of hydrogen peroxide (H_2_O_2_ 100 mM), reaching a final volume of 300 µL per well. Catalase activity was determined by measuring the decrease in absorbance at 240 nm wavelength due to H_2_O_2_ decomposition, using a Synergy HTX microplate reader (Biotek, Santa Clara, CA, USA). Readings were taken every 15 s for 5 min in kinetic mode. Enzyme activity was expressed as units of catalase per milligram of protein (U/mg protein) [34,35].

### 2.9. Determination of Protein Concentration

Protein content in brain homogenates was determined using a modified Lowry method. Briefly, 10 µL of homogenate was diluted using 190 µL of deionized water. Then, 1 mL of solution C (a mixture of solution A (2% Na_2_CO_3_, 0.4% NaOH, 0.2% sodium-potassium tartrate) and solution B (0.5% CuSO_4_) was added and incubated for 10 min at room temperature. Next, 100 µL of 50% Folin reagent was added, followed by a 30-minute incubation at room temperature. Absorbance was measured at 550 nm wavelength using a Sinergy H1 microplate reader (Agilent, Santa Clara, CA, USA). Protein concentrations were determined using a BSA standard curve.

### 2.10. Reverse Transcription (RT) and Quantitative PCR (qPCR)

200 μL of TRIzol reagent (ThermoFisher: 15596026, Waltham, MA, USA.) was used to extract total RNA from rat striatum, hippocampus, or cerebral cortex. RNA concentration was determined with a Take3 microvolume plate (Agilent: TAKE3-SN) coupled to a BioTek Synergy H1 multimode microplate reader (Agilent: 11-120-533). Then, 500 ng of total RNA were reverse transcribed as previously described [36]. Obtained complementary DNAs (cDNAs) were diluted in sterile water at 1:5 and stored at −20 °C until use. 5 μL qPCR reactions were prepared by mixing 2.25 μL of the corresponding diluted cDNA, 2.5 μL of the 2X Brilliant III Ultra-Fast SYBR Green qPCR Master Mix with Low ROX (Agilent: 600892) and 0.25 μL of 10 µM oligo mix to amplify either the *Nrf2* gene (rat transcript ENSRNOT00055035614.1 in Ensembl; Fwd: GAAGGAACAGGAGAAGGCC, Rev: TCTTGTTTGGGAATGTGGG) or the β-actin one (rat transcript ENSRNOT00000065528.2 in Ensembl; Fwd: CGTGCGGGATGTCAAAGAA, Rev: AACGTTCGTTCCCAATGGTG) with a melting temperature (Tm) of 60 °C and 40 cycles. All qPCRs were performed in an AriaMx real-time Thermalcycler (Agilent: G8830A). The relative expression level of *Nrf2* (normalized to β-actin) was calculated using the 2^−ΔΔCt^ method.

### 2.11. Quinolinic Acid Intrastriatal Administration and Circling Behavior

A total of 20 male Wistar rats were randomly divided into four experimental groups (n = 5 per group). Control (received oral vehicle and intrastriatal saline); Hops group (treated with Hops extract (15 mg/kg/day, p.o.) and intrastriatal saline), QUIN group (received oral vehicle and intrastriatal QUIN), and Hops + QUIN group (pretreatment with Hops (10 mg/kg/day, p.o. and intrastriatal QUIN). Hops extract was administered once daily for 7 days prior to intrastriatal surgery. On day 7, animals were anesthetized with xylazine (10 mg/kg i.p.) and ketamine (50 mg/kg i.p.), and a single unilateral intrastriatal injection of either saline (1 µL) or QUIN (240 nmoles/µL) was performed following stereotaxic coordinates (AP +0.5 mm from bregma, L +3.0 mm from bregma and V −4.6 mm from dura). To assess the degree of dopaminergic dysfunction induced by intrastriatal QUIN, circling behavior was evaluated five days post-surgery. Rats were administered apomorphine (1 mg/kg s.c.), and animals were placed individually into acrylic observation chambers; after a 10-min acclimation period, the number of full ipsilateral rotations was manually recorded for 30 min. Behavioral scoring was performed blinded to group allocation. Results were expressed as the number of complete turns per 30 min, following a previously established protocol [37].

### 2.12. Apoptosis Determination

Apoptosis was quantified in striatal tissue using the Cell Death Detection ELISA PLUS kit (Roche, Cat. N. 11920685001, Mannheim Germany), according to the manufacturer’s instructions. This method is based on the quantitative detection of histone-associated DNA fragments in the cytoplasmic fraction of tissue lysates. Briefly, striata were dissected from rats that received intrastriatal QUIN or saline, with or without Hops extract pre-treatment. Tissue samples were homogenized, lysed, and centrifuged, and supernatants were used for ELISA detection. Absorbance was measured using a Sinergy H1 microplate reader (Agilent, Santa Clara, CA, USA) at 405 nm wavelength. Apoptosis levels were expressed as a percentage relative to the control group.

### 2.13. Cell Fractionation

30 mg of brain tissue (striatum) were homogenized in 3 volumes of buffer A (10 mM HEPES, 10 mM KCl, 1mM EDTA, 0.4% Igepal, 1 mM Na_3_VO_4_, 1 mM DTT, 1 mM PMSF, and protease inhibitor cocktail) using a tissue homogenizer. Cell suspensions were centrifuged at 4000× *g* at 4 °C for 15 min. Supernatants (soluble cytoplasmic fractions) were collected in new tubes and stored at −80 °C until use. Pellets were washed with buffer A and spun at 4000× *g* at 4 °C for 15 min twice. After washing, supernatants were discarded, and pellets were resuspended in 100 μL of buffer B (20 mM HEPES, 200 mM NaCl, 1mM EDTA, 5% glycerol, 1mM Na_3_VO_4_, 1mM DTT, 1 mM PMSF, and protease inhibitor cocktail) and incubated on ice for 2 h. After incubation, samples were centrifuged at 15,000× *g* 4 °C for 15 min, and supernatants (soluble nuclear fractions) were collected and stored at −80 °C until use. Protein concentration was determined as described above.

### 2.14. Western Blot

NRF2 protein levels were determined by Western blot in both striatum-derived nuclear and cytoplasmic fractions. Briefly, either 50 μg of cytoplasmic or 20 μg of nuclear extracts were separated into 8% SDS-PAGE gels and transferred onto nitrocellulose membranes. Membranes were blocked using 3% BSA for 1 h and incubated with an anti-NRF2 HRP conjugated antibody (sc-518036, Santa Cruz Biotechnology, CA, USA) at a dilution of 1:1000 overnight at 4 °C. Next day, membranes were washed three times using PBS + 0.5% Tween 20 (PBS-T) at room temperature for 10 min and then, protein bands were detected using the NZY Advanced ECL Chemiluminiscent Substrate for Western blotting (MBA 40201, NZYtech, Lisbon, Portugal). For this, membranes were imaged using the UVP Chemstudio (Analitikena, Jena, Germany) and analyzed using the ImageJ software 1.54 g (US National Institutes of Health, Bethesda, MD, USA). Relative protein levels were determined by normalizing the optical density of each band by the signal obtained for its corresponding Ponceau signal as a loading control. Membranes were incubated overnight at 4 °C using either an anti-β-Actin antibody at a 1:500 dilution (sc-130300, Santa Cruz Biotechnology, Paso Robles, CA, USA) or an anti-Lamin B1 antibody at a 1:2000 dilution (AB133741, Abcam, Cambridge, UK) to confirm the correct fractionation of cytoplasmic and nuclear samples, respectively. After that, membranes were incubated with their respective secondary antibodies for 1 h, washed three times, and revealed as previously described.

### 2.15. Statistical Analysis

Data are presented as the mean ± standard error of the mean (SEM). Statistical analyses were performed using GraphPad Prism 7 software (GraphPad software, San Diego, CA, USA). For comparisons involving more than two independent groups, data were analyzed using the Kruskal–Wallis test, followed by Dunn’s tests for multiple comparisons. When comparing only two groups, a Mann–Whitney test was performed. *p*-values lower than *p* < 0.05 were considered statistically significant.

## 3. Results

Hops are known to contain various antioxidant compounds that have demonstrated neuroprotective effects in different experimental models. However, most studies have focused on isolated constituents, which can be costly to extract and often require high concentrations to achieve significant effects. Therefore, we aimed to evaluate whether a complete Hops extract, without its separation into individual components, could exert and provide antioxidant activity and neuroprotection.

### 3.1. Hops Extract Acts as a Reactive Oxygen Species (ROS) Scavenger on Combinatorial Chemistry Assays

**Figure 1:**
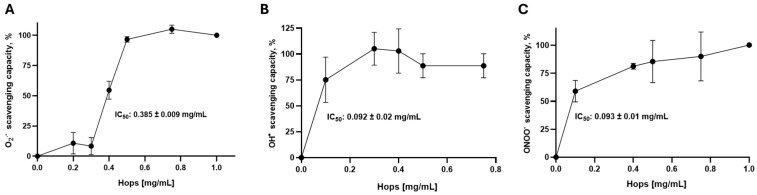
Scavenging activity of Hops extract (0–1 mg/mL) against (**A**) superoxide anion (O_2_^●^^−^), (**B**) hydroxyl radical (●OH), and (**C**) peroxynitrite (ONOO^−^), evaluated by using combinatorial chemical-based assays. Data are presented as the mean ± S.E.M. of 5–6 independent experiments per concentration.

To evaluate the redox potential of the Hops extract, its ability to scavenge biologically relevant reactive oxygen species (ROS) was first assessed. Since the superoxide anion (O_2_^●^^−^), hydroxyl radical (●OH), and peroxynitrite (ONOO^−^) play a central role in cellular signaling and oxidative damage, these ROS were used to evaluate the radical scavenging activity of the hops extract through combinatorial chemistry-based assays. As shown in Figure 1, the Hops extract neutralized all three tested ROS, with the greatest efficacy observed against ●OH and ONOO^−^. The half-maximal inhibitory concentration (IC_50_) values for ●OH and ONOO^−^ were 0.092 ± 0.02 mg/mL and 0.093 ± 0.01 mg/mL, respectively. In contrast, a significantly higher concentration was required to scavenge superoxide, with an IC_50_ of 0.385 ± 0.009 mg/mL, indicating approximately a four-fold lower potency for this ROS.

**Figure 2:**
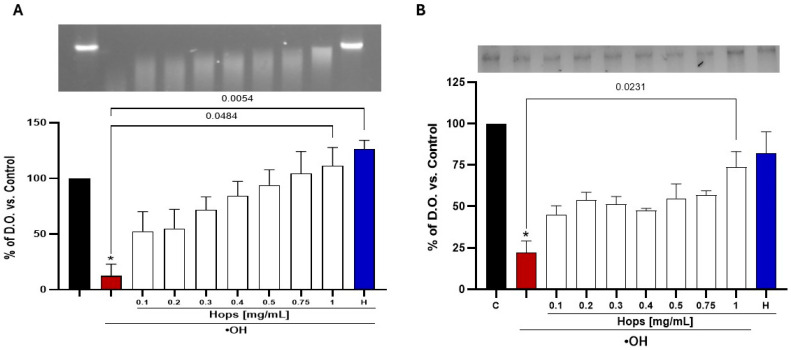
Protective effect of Hops extract against (**A**) DNA and (**B**) protein oxidative degradation induced by a •OH generation system. Black bars represent untreated DNA or protein (negative controls); red bars represent samples exposed only to the •OH generating system (positive controls); white bars indicate samples (DNA or protein) co-treated with the •OH generation system and increasing concentrations of the Hops extract; blue bars represent samples treated with highest tested concentration (1 mg/mL) of the used Hops extract. Data represent the mean ± S.E.M. of 3 independent experiments. * and *p*-values based on the Kruskal–Wallis test, with Dunn’s test for pairwise comparisons.

Next, the capacity of the Hops extract for preventing oxidative damage to biomolecules was evaluated. Specifically, the ability of the Hops extract to inhibit ●OH radical-induced DNA and protein degradation in a cell-free system was assessed. Hydroxyl radicals are among the most reactive ROS and are known to directly fragment DNA and oxidize protein side chains, leading to structural and functional impairment. As shown in Figure 2A,B, the Hops extract showed a concentration-dependent trend towards reducing both DNA and protein degradation. However, a statistically significant protective effect was only observed at the highest tested Hops extract concentration (1 mg/mL).

### 3.2. Effect of Subchronic Administration of Hops Extract on Brain Glutathione Status

After confirming that the used Hops extract possesses redox-modulating properties and protects biomolecules in cell-free chemical assays, its antioxidant effects in an in vivo model were tested. For this purpose, three different doses of the Hops extract (10, 15, and 20 mg of Hops/kg/day) were administered orally to rats for 7 consecutive days. Subsequently, brains were collected, and levels of reduced (GSH) and oxidized (GSSG) glutathione were measured, as glutathione is a major endogenous non-enzymatic antioxidant known to reflect redox balance in brain tissue.

**Figure 3:**
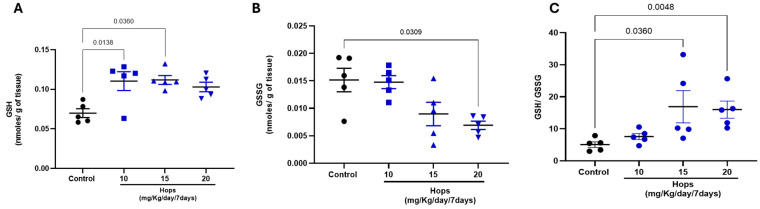
Effect of subchronic oral administration of Hops extract on brain levels of (**A**) reduced glutathione (GSH), (**B**) oxidized glutathione (GSSG), and (**C**) GSH/GSSG ratio. Rats were administered at doses of 10, 15, and 20 mg/kg/day Hops extract for 7 days. Data are expressed as the mean ± S.E.M. (n = 5). *p*-values are based on the Kruskal–Wallis test followed by Dunn’s tests.

As shown in Figure 3, brain GSH levels increased significantly after Hops extract’s administration at all three doses, nearly doubling their levels when compared to control values. The observed GSH concentrations were 0.110 ± 0.01, 0.120 ± 0.005, and 0.103 ± 0.005 nmoles per g of tissue for doses 10, 15, and 20 mg of Hops/kg/day, respectively, vs. 0.070 ± 0.005 nmoles/g in tissue of the control group. In addition, brain GSSG levels significantly decreased only at the 15 and 20 mg/kg doses (being 0.009 ± 0.002 and 0.007 ± 0.001 nmol/g tissue, respectively), compared to 0.015 ± 0.002 nmoles/g in brain tissue of the control group. Importantly, when the GSH/GSSG ratio was calculated, a robust and statistically significant increase was observed in the tissue of 15 and 20 mg/kg Hops-administered groups, indicating a shift toward a more reduced (antioxidant-favorable) intracellular environment in the brain when compared to the control group.

**Figure 4:**
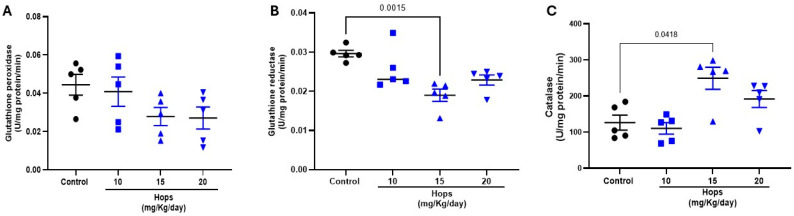
Effect of subchronic oral administration of Hops extract on the activity of (**A**) glutathione peroxidase (GPx), (**B**) glutathione reductase (GR), and (**C**) catalase in the brain. Rats were treated with 10, 15, and 20 mg/kg/day of Hops extract for 7 days. Data are expressed as the mean ± S.E.M. (n = 5). *p*-values based on the Kruskal–Wallis test followed by Dunn’s tests.

To further explore the mechanisms underlying the enhanced redox environment observed after Hops extract administration, the activity of enzymes involved in glutathione metabolism, glutathione peroxidase (GPx), and glutathione reductase (GR) was determined in brain tissue. As shown in Figure 4A, GPx exhibited a trend to decrease in both 15 and 20 mg/kg Hop’s administered groups (0.028 ± 0.005 and 0.027 ± 0.006 U/mg protein, respectively) when compared to the control one (0.044 ± 0.0054 U/mg protein), although the differences were not statistically significant. While GR activity was significantly reduced in the 15 mg/kg Hops extract-treated group (0.019 ± 0.002 U/mg protein) when compared to the control one (0.030 ± 0.001 U/mg protein) (Figure 4B), suggesting that the antioxidant environment promoted by the extract may reduce the cellular demand and the activity of antioxidant enzymes. Additionally, the activity of catalase, a key antioxidant enzyme involved in the detoxification of hydrogen peroxide (H_2_O_2_), was assessed in brain tissue. As shown in Figure 4C, administration of Hops extract at doses of 15 and 20 mg/kg produced an increase in catalase activity compared to the control. Notably, the 15 mg/kg dose induced a statistically significant enhancement in catalase activity (249 ± 30 U/mg protein vs. 126 ± 21 U/mg protein), suggesting that this dose may effectively boost the endogenous antioxidant defense. These results further support the notion that Hops extract modulates enzymatic components of the redox system, complementing the observed effects on GSH-related pathways.

### 3.3. Ex Vivo Pro-Oxidant Challenge Following Hops Extract Administration

**Figure 5:**
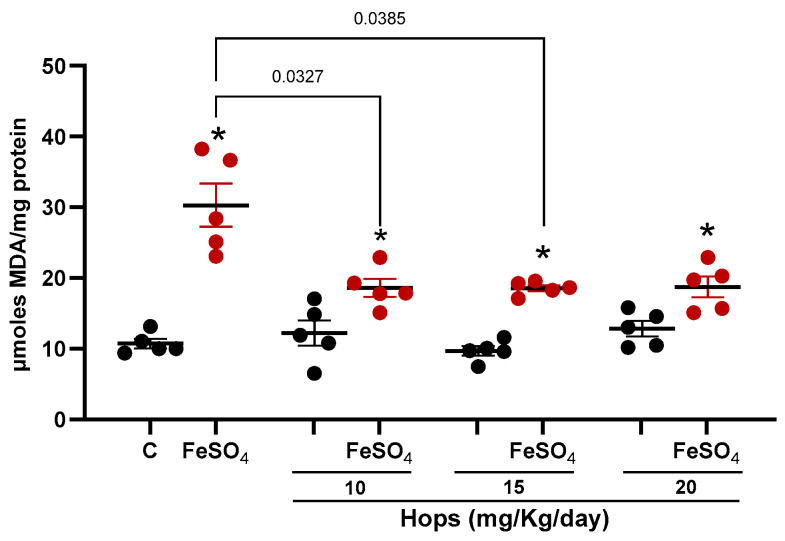
Ex vivo lipid peroxidation in brain tissue following subchronic Hops administration and exposure to FeSO_4_. Rats were administered doses of 10, 15, and 20 mg/kg/day of the Hops extract for 7 days. Brain tissue was then collected and incubated ex vivo with 100 µM FeSO_4_ to induce lipid peroxidation. Black dots: without FeSO_4_ exposure, and red dots: FeSO_4_ exposure. Data are expressed as the mean ± S.E.M. (n = 5). * *p*-value is based on the Mann–Whitney test when compared to the respective control. Numeric *p*-values are based on the Kruskal–Wallis test followed by Dunn’s tests comparing all groups treated with FeSO_4_.

After confirming that Hops extract enhances the antioxidant status of the brain in vivo, the next step was to determine whether this biochemical shift translated into improved resistance to oxidative damage under pro-oxidant conditions. This is particularly relevant in the central nervous system, as the brain is highly enriched in polyunsaturated fatty acids (PUFAs), which are essential components of neuronal membranes. These PUFAs are highly susceptible to lipid peroxidation, making oxidative damage to membrane lipids a critical event in the pathogenesis of various neurodegenerative diseases. Actually, lipoperoxidation not only compromises membrane integrity and fluidity but also generates reactive aldehydes that can further damage biomolecules and disrupt cellular signaling and integrity [38]. Therefore, brain tissue from all experimental groups was collected and exposed to FeSO_4_ ex vivo, a well-established inducer of lipid peroxidation. As shown in Figure 5, exposure to FeSO_4_ significantly increased lipid peroxidation levels in all groups, as measured by standard oxidative stress markers. However, the magnitude of lipoperoxidation varied markedly between groups. Briefly, in the control group, FeSO_4_ -exposure resulted in approximately a 200% increase compared to its non-exposed baseline. In contrast, tissue obtained from Hops-treated animals showed a significantly attenuated response, reaching only about 50% of that observed in their respective untreated controls. Importantly, levels of lipid peroxidation in FeSO_4_-exposed tissue from all Hops-treated groups were significantly lower than those observed in the FeSO4-exposed control group, indicating that the subchronic administration of the Hops extract enhanced the brain’s resilience to oxidative insults.

### 3.4. Hops Extract Administration Promotes an Antioxidant Environment and Modulates Nrf2 Expression in Brain Regions

Based on the observed enhancement in the GSH/GSSG ratio and the attenuation of FeSO_4_-induced lipid peroxidation, the ability of the Hops extract to modulate basal lipid peroxidation in distinct brain regions was examined. This approach aimed to determine whether the protective effects of Hops exhibit regional specificity in the brain. As shown in Table 1, a consistent trend toward decreased basal lipid peroxidation across all the evaluated brain regions was observed. Notably, both the striatum and the hippocampus exhibited the most pronounced reductions in lipid peroxidation (30–35% compared to the control), while in the cortex, the maximal reduction was around 25% compared to its respective control.

**Figure 6:**
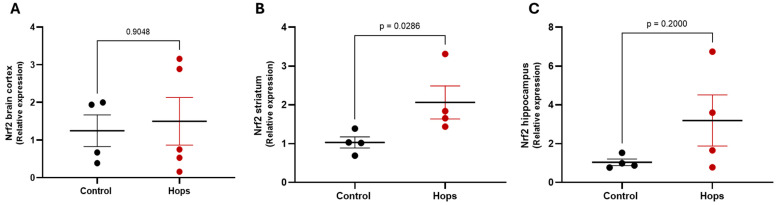
Effect of subchronic Hops administration on *Nrf2* expression in different brain regions. Relative expression levels of Nrf2 were determined by quantitative PCR in the (**A**) cortex, (**B**) striatum, and (**C**) hippocampus. Data are expressed as the mean ± S.E.M. (n = 4–5). *p*-values are based on the Mann–Whitney test.

After these observations, the 15 mg/kg/day dose of Hops was selected for further molecular analysis. Thus, to explore a possible mechanism underlying the antioxidant effect of Hops, the expression of the *Nrf2* gene, a master transcriptional regulator of redox homeostasis and cellular defense mechanisms, was assessed in three brain regions: cortex, striatum, and hippocampus by quantitative PCR (qPCR). As shown in Figure 6, *Nrf2* expression levels were significantly increased in the striatum of Hops-treated animals compared to controls. Moreover, while the cortex did not show significant changes, the hippocampus exhibited an upward trend in *Nrf2* expression, though it did not reach statistical significance. These results suggest that Hops may exert its neuroprotective effects, at least in part, by transcriptionally activating NRF2-regulated antioxidant defense systems in the striatum and possibly in the hippocampus.

### 3.5. Effects of Hops Extract on Quinolinic Acid-Induced Excitotoxicity and Apoptosis in the Striatum

**Figure 7:**
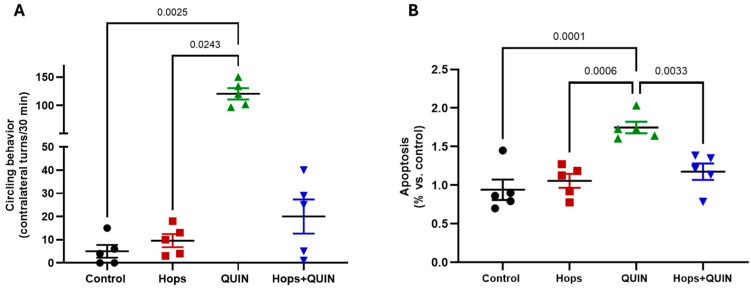
Effect of Hops pre-treatment (15 mg/kg/day for 7 days) on the excitotoxicity induced by QUIN. (**A**) Ipsilateral rotational behavior induced by apomorphine as a marker of QUIN-induced striatal damage. (**B**) Quantification of apoptotic cell death in striatal tissue. Data are expressed as mean ± S.E.M. (n = 5). *p*-values are based on the Kruskal–Wallis test followed by Dunn’s tests.

Given the significant upregulation of *Nrf2* in the striatum following Hops treatment, we investigated whether this molecular change translated into functional neuroprotection in a model of excitotoxicity. For this purpose, quinolinic acid (QUIN), a well-characterized *N*-methyl-D-aspartate (NMDA) receptor agonist known to induce excitotoxicity and neuronal death, particularly in brain regions rich in GABAergic neurons such as the striatum, was employed. Briefly, QUIN was injected into the striatum to induce localized neurodegeneration. To assess functional damage, rotational behavior induced by apomorphine, which reflects asymmetrical striatal injury, was determined. As shown in Figure 7A, animals in the QUIN group showed a marked increase in ipsilateral rotations (120.6 ± 9.9 turns) compared to the control group (5 ± 2.7 turns). Notably, animals pre-administered with the Hops extract (15 mg/kg/day) for 7 days prior to QUIN injection (Hops + QUIN group) exhibited an 83.4% reduction in turning behavior relative to the QUIN group (20.0 ± 7.4 turns), although this reduction did not reach statistical significance.

In parallel, we assessed apoptotic cell death in the striatal tissue across all experimental groups. As shown in Figure 7B, QUIN administration significantly increased the level of apoptosis (85.9% vs. control). Importantly, Hops pre-treatment reduced apoptosis by approximately 33% compared to the QUIN group. These results suggest that the Hops extract could confer partial neuroprotection against QUIN-induced excitotoxicity, potentially through the modulation of oxidative and apoptotic pathways in the striatum.

**Figure 8:**
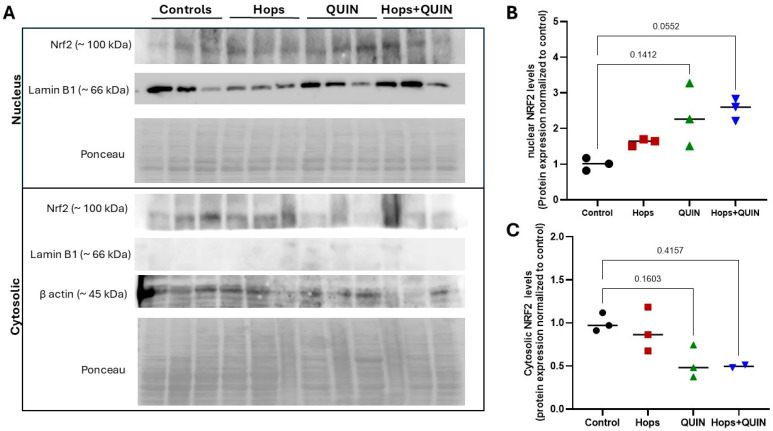
Hops extract modulates NRF2 protein levels in nuclear and cytosolic brain-derived cell fractions. (**A**) Representative Western blot images showing NRF2 protein levels in nuclear and cytosolic fractions obtained from the striatum of rats treated with vehicle (Control), Hops extract (15 mg/kg), QUIN, or their combination (Hops + QUIN). Ponceau staining was used as a protein loading control, and β-actine and lamin B1 were used as positive controls to verify the correct isolation of the cytosolic and nuclear fractions, respectively. Densitometric analysis of NRF2 protein levels is shown for (**B**) nuclear and (**C**) cytosolic fractions (n = 3 animals per group). Data are expressed as the mean ± SEM. *p*-values are based on the Kruskal–Wallis test followed by Dunn’s tests.

To further investigate the mechanism underlying the protective effects of Hops extract, the protein levels of the transcription factor NRF2 were evaluated in cytosolic and nuclear fractions from the striatum. As shown in Figure 8, Hops administration alone did not significantly alter cytosolic NRF2 levels, although a slight increase was observed in the nuclear fraction, suggesting a modest trend toward NRF2 activation by its nuclear translocation. In the QUIN-treated group, a moderate increase in nuclear NRF2 levels was detected, accompanied by a non-significant decrease in the cytosolic fraction, consistent with a partial activation of the NRF2 pathway in response to excitotoxic stress. Notably, in the Hops + QUIN group, a further decrease in cytosolic NRF2 levels was observed, together with a strong upward trend in the nuclear fraction, indicating enhanced nuclear translocation. This pattern supports the idea that subchronic Hops administration may prime NRF2 activation and promote its nuclear accumulation upon a subsequent insult, thereby contributing to the observed antioxidant and neuroprotective effects.

## 4. Discussion

Oxidative stress and excitotoxicity are recognized as central pathological mechanisms in the development and progression of several neurodegenerative diseases, including Alzheimer’s disease, Parkinson’s disease, and amyotrophic lateral sclerosis [39,40,41]. These mechanisms converge through excessive production of ROS, disruption of antioxidant defense systems, mitochondrial dysfunction, and ultimately, neuronal cell death [40,41]. Beyond their involvement in neurodegenerative pathology, oxidative stress also plays a key role in normal aging, contributing to a gradual decline in mitochondrial function, synaptic plasticity, and cognitive performance [42]. As the global incidence of age-related neurological disorders continues to rise, there is a growing interest in identifying natural compounds capable of modulating redox balance and enhancing neuronal resilience. In this context, plant-derived compounds are being actively explored as promising candidates for preventive strategies.

This study aimed to investigate whether a non-fractionated extract of *Humulus lupulus* (Hops) could provide such neuroprotective effects, leveraging the synergistic action of its bioactive compounds. While a substantial body of literature has already described the antioxidant and anti-inflammatory properties of isolated prenylflavonoids such as xanthohumol and bitter acids from Hops [17,25,43,44], less is known about the efficacy of the whole extract, which is more accessible, cost-effective, and potentially suitable for nutraceutical or preventive clinical applications. These findings support the idea that the full Hops extract retains potent biological activity, possibly due to synergistic interactions between polyphenols, bitter acids, and other Hops-derived compounds. Notably, the used Hops extract demonstrated robust free radical scavenging activity against superoxide, hydroxyl radical, and peroxynitrite. The IC_50_ values for the hydroxyl radical and the peroxynitrite were comparable, indicating a similar degree of efficacy against these highly reactive species. Although the extract was also effective against superoxide, a higher concentration (~3 times greater) was required to achieve comparable scavenging activity of this ROS. These findings are consistent with the results obtained with the performed chemical combinatorial assay, which showed that the Hops extract effectively protected both DNA and proteins from degradation induced by hydroxyl radicals. These results extend previous studies where xanthohumol was shown to protect DNA from strand breaks and inhibit protein oxidation in vitro [43,45,46].

Importantly, oral administration of the Hops extract increased brain GSH levels and decreased GSSG, leading to a significantly improved GSH/GSSG ratio, which is considered a hallmark of improved redox homeostasis. These changes reflect an increased intracellular detoxification capacity and thiol buffering, which are essential mechanisms for neuron protection from cumulative oxidative damage [15,47,48]. Supporting this redox improvement, a reduction in basal lipid peroxidation was also observed, particularly in the striatum and the hippocampus, which are highly susceptible to oxidative stress. These changes further indicated a region-specific enhancement of the redox environment in the brain. Moreover, the activity of both GPx and GR was found to be reduced following Hops extract administration, which could reflect a decreased cellular demand for detoxifying ROS, consistent with the establishment of a more favorable redox environment induced by the Hops extract used. The observed resilience to the evaluated ex vivo oxidative challenge (FeSO_4_) further reinforces the idea that Hops pretreatment enhances the brain’s ability to buffer oxidative insults. Such adaptive conditioning may involve both direct antioxidant effects and indirect upregulation of endogenous defenses, as previously proposed for polyphenol-rich botanicals like green tea and curcumin [49,50,51].

Given the known role of xanthohumol in activating NRF2, a master transcription factor orchestrating the antioxidant response, we examined whether Hops extract also modulates *Nrf2* expression in the brain. Our data revealed a significant upregulation of *Nrf2* in the striatum, and a non-significant upward trend in the hippocampus. NRF2 promotes the transcription of cytoprotective genes such as *Hmox1*, *Nqo1*, *Gclc*, *Txn*, and *Trxr1*, which collectively enhance both antioxidant capacities and stress tolerance of cells [47,52,53]. Interestingly, the regional specificity of *Nrf2* induction (being most evident in the striatum) may reflect differential bioavailability or metabolic activation of Hops extract components across brain regions. Alternatively, this pattern could be attributed to inherent differences in the basal levels of oxidative stress or redox-sensitive signaling pathways between the striatum and the hippocampus. Such region-dependent responsiveness has been previously reported in studies involving dietary polyphenols and pharmacological NRF2 activators, suggesting that tissue-specific redox tone may influence the magnitude of transcriptional adaptation [49,54,55].

After observing a significant upregulation of *Nrf2* expression in the striatum, whether this molecular change is translated into a functional enhancement of cellular resilience against injury was explored. Given the well-established role of NRF2 in regulating antioxidant and cytoprotective pathways [56], it was hypothesized that the increase in *Nrf2* expression induced by the Hops extract could contribute to mitigating neuronal vulnerability to excitotoxicity. In this regard, a model using quinolinic acid (QUIN), which mimics glutamate-induced neurodegeneration via overactivation of NMDA receptors, was employed. In this model, mitochondrial dysfunction, calcium overload, ROS production, and eventual neuronal death occur [57]. Interestingly, pre-treatment with the Hops extract attenuated circling behavior induced by QUIN, as an indicator of functional asymmetry resulting from striatal damage. This behavioral improvement is consistent with the observed reduction in neuronal apoptosis, suggesting that the Hops extract confers both structural and functional neuroprotection. This protective effect may be mediated, at least in part, by the enhanced translocation of NRF2 to the nucleus, as observed in the protein levels analysis. The nuclear localization of NRF2 is known to trigger the transcription of its target genes, including various antioxidant and cytoprotective genes, which likely contribute to the attenuation of excitotoxic damage and preservation of neuronal integrity. While similar effects have been attributed to xanthohumol in the kainic acid-induced excitotoxic model [58], the findings of this study are notable because a lower and more sustained dose of the full extract was employed, emphasizing both its efficacy and translational feasibility.

The concept of promoting redox resilience, defined as the ability of cells to withstand and to adapt to oxidative perturbations, is increasingly recognized as a preventive strategy against neurodegeneration [59]. Unlike approaches that merely scavenge ROS, redox resilience implies the enhancement of adaptive cellular responses, such as those governed by NRF2 and mitochondrial function [60,61]. In this regard, this study positions the complete Hops extract as a promising candidate among natural compounds able to induce redox resilience. Its low cost, oral bioavailability, and multimodal effects make it an attractive candidate for nutritional intervention targeting aging in populations or individuals at risk of developing neurodegenerative disorders.

Some limitations of this study must be addressed. The major limitation is the absence of a complete chemical characterization of the Hops extract used, as well as the lack of comparison with the effects of individually purified reference compounds. Such additional studies would help to clarify the relative contribution of individual constituents versus the potential synergistic effects of the whole extract. Additionally, longer treatment durations and further dose optimization may reveal more robust neuroprotective outcomes and, consequently, provide deeper mechanistic insights. Nonetheless, findings in this work lay a foundation for using the complete Hops extract as a neuroprotective compound, supporting its use in preclinical models of oxidative stress and excitotoxicity, and encouraging further exploration of its potential in preventing brain aging and neurodegeneration.

## 5. Conclusions

Taken together, the findings of the present research support that the subchronic administration of a full Hops extract promotes a redox-adaptative state in the brain, enhancing its ability to withstand both oxidative and excitotoxic insults. Given the increasing burden of age-related neurodegenerative diseases, strategies that foster neuroresilience through natural, accessible compounds may represent a promising direction for preventive interventions. Further studies should explore its long-term efficacy, optimize dosing regimens, and evaluate its translational potential in clinical settings.

## Figures and Tables

**Table 1:** Effect of Hops extract administration on basal lipid peroxidation in different brain regions.

		Hops Extract (mg/kg/day)		
Lipid Peroxidation (µmol MDA/mg Protein)	Control	10	15	20
Cortex	18.94 ± 2.4	18.29 ± 2.1	17.75 ± 2.9	15.87 ± 4.2
Striatum	19.50 ± 3.1	13.02 ± 0.9	11.54 ± 3.0	12.56 ± 3.3
Hippocampus	19.85 ± 2.9	14.04 ± 1.6	12.77 ± 1.5	12.52 ± 1.4

Data are expressed as the mean ± S.E.M. (n = 5). Based on the Kruskal–Wallis test followed by Dunn’s tests.

## Data Availability

The data presented in this study are available on request from the corresponding author due to the responsible use of the data.

## Abbreviations

The following abbreviations are used in this manuscript:

●OH	Hydroxyl radical
BSA	Bovine serum albumin
CaCl_2_	Calcium chloride
cDNAs	Complementary DNAs
CuSO_4_	Copper (II) sulfate
DCF	Dichlorofluorescein
DCFH-DA	Dichlorofluorescein diacetate
DFO	Deferoxamine
DNA	Deoxyribonucleic acid
DTPA	Diethylene triamine pentaacetic acid
DTT	Dithiothreitol
EDTA	Ethylenediaminetetraacetic acid
FeCl_3_	Ferric chloride
FeSO_4_	Ferrous sulfate
GSH	Reduced glutathione
GSSG	Oxidized glutathione
H_2_O_2_	Hydrogen peroxide
Hops	Humulus lupulus
IL-6	Interleukin-6
MDA	Malondialdehyde
NBT	Nitroblue tetrazolium
NEM	*N*-ethylmaleimide
NF-κB	Nuclear factor kappa B
NMDA	*N*-methyl-D-aspartate
Nrf2	Nuclear factor erythroid 2-related factor
O_2_^●^^−^	Superoxide anion
ONOO^−^	Peroxynitrite
OPA	O-pthaldehyde
QUIN	Quinolinic acid
ROS	Reactive oxygen species
